# Deciphering Pain and Pruritus in Keloids from the Perspective of Neurological Dysfunction: Where Are We Now?

**DOI:** 10.3390/biomedicines13030663

**Published:** 2025-03-08

**Authors:** En Yang, Ruoqing Xu, Hanrui Zhang, Wenzheng Xia, Xin Huang, Tao Zan

**Affiliations:** Department of Plastic and Reconstructive Surgery, Shanghai Ninth People’s Hospital, School of Medicine, Shanghai Jiao Tong University, Shanghai 200021, China; doc.enyang@gmail.com (E.Y.); annahsu@163.com (R.X.); zhrui0702@163.com (H.Z.); wisdomzheng@126.com (W.X.)

**Keywords:** keloid, pruritus, pain, neurological dysfunction, Schwann cell

## Abstract

Keloids are a typical skin fibroproliferative disease that can cause severe aesthetic and functional concerns. Pain and pruritus are the most common clinical symptoms of keloids, but the mechanisms underlying these symptoms remain unclear. The peripheral nervous system plays a pivotal role in the transmission of superficial sensation signals. Mounting evidence has shown potential correlations between disturbance in the peripheral nervous system and pain and pruritus in keloids. Here, we summarize the role of neurological dysfunction in the development of pain and pruritus, with a specific focus on neuroanatomical alterations, the dysfunction of sensory nerves, and neurogenic inflammation.

## 1. Introduction

Keloids are fibroproliferative dermal tumors characterized by excessive collagen deposition and infiltrative growth beyond the initial injury margin [1]. The dysregulation of the wound-healing process in keloids is marked by persistent inflammation, excessive fibroblast activation, and an imbalance between collagen synthesis and degradation. This abnormal healing is characterized by an overproduction of type I and III collagen, leading to dense, fibrotic tissue. Genetic factors and altered molecular pathways, such as the TGF-β/Smad, JAK/STAT, and PI3K/AKT pathways, play significant roles in keloid formation, with mechanical forces further exacerbating fibrosis by enhancing fibroblast proliferation and ECM deposition [2]. With the advancement of single-cell sequencing technologies, previous studies have highlighted comprehensive microenvironmental intercellular crosstalk between fibroblasts and endothelial cells, immune cells, and Schwann cells in the pathogenesis of keloids [3,4,5].

Keloids not only lead to severe aesthetic and functional concerns, but also cause frustrating sensory discomfort, including pain and pruritus. Approximately 50–90% of keloid patients suffer from pain or itching [6,7]. However, the mechanism underlying the development of pain and pruritus remains unknown. These symptoms have been reported to be related to inflammatory activity during keloid progression, including the activation of immune cells [8,9] (mast cells, T cells, and macrophages) and the release of inflammatory compounds [10,11] (histamines, interleukin [IL]-4, IL-13, and IL-31). These inflammatory mediators indirectly or directly stimulate nerve fibers, inducing pain and pruritus in keloids. Therefore, antihistamine drugs and corticosteroids are commonly used for the control of keloid-associated pain and pruritus [12]. However, dysfunction of the inflammatory microenvironment is only one factor. Even with the use of the first-line drug triamcinolone acetonide, a significant proportion of patients will not experience improvements in their functional symptoms [13].

**Figure 1:**
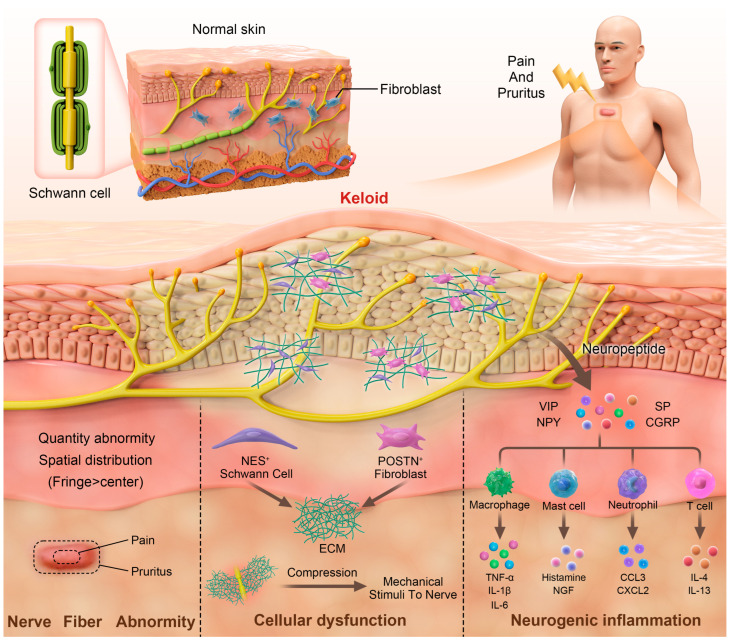
A putative mechanism of pain and pruritus in keloids. Neurological dysfunction in keloids includes abnormalities of nerves within the keloid, cellular activation leading to nerve damage, and neurogenic inflammation. ECM: extracellular matrix; NPY: neuropeptide Y; VIP: vasoactive intestinal peptide; SP: substance P; CGRP: calcitonin gene-related peptide; TNF: tumor necrosis factor; NGF: nerve growth factor; CCL: C-C motif chemokine ligand; CXCL: C-X-C motif chemokine ligand; IL: interleukin.

Another factor in the etiology of keloid patients may be related to neurological dysfunction. Skin pain and pruritus are meticulously controlled by several kinds of sensory nerve fibers (mainly Aδ and C fibers) [14,15]. In cases of neural injury, primary and secondary afferent nerve fiber activation may release inflammatory mediators that potentially activate pruriceptors [16]. Since the pathogenesis of keloids begins with cutaneous-injury-induced nerve damage and abnormal wound healing machinery, neurological dysfunction factors definitely play a role in the formation of pain and itch in keloid patients to a certain degree. Here, we summarize the currently available evidence and try to elucidate the underlying mechanisms of keloid-associated pain and pruritus involving neurological dysfunction (Figure 1).

## 2. Neuroanatomical Alterations and Dysfunction of Sensory Nerves

Generally, abundant nerve fibers, including autonomic and sensory nerves, are densely distributed throughout all layers of the skin. Sensory nerves branch out like dendrites, forming free nerve endings and various types of corpuscles, such as Pacinian and Ruffini corpuscles [14,17]. Therefore, abnormalities in the density or morphology of the nerves often lead to abnormal sensation [18,19].

However, the quantity and spatial distribution of nerve fibers in keloid patients are still controversial. Hochman et al. performed an S100 immunohistochemical analysis of keloid and normal skin [20]. The results showed that, compared with normal skin, more S100-positive nerve fibers are located in the dermis of keloids. In contrast, Tey et al. [21] and Saffari et al. [22] observed decreased nerve fibers in both the epidermis and upper dermis of keloids. The reason for this inconsistency is unclear and may be related to various factors. First, the selection of markers used to visualize nerves is critical. S100 is widely expressed in epidermal cells, such as Schwann cells, melanocytes, and Langerhans cells (LCs) [23], and the use of S100 as a nerve fiber marker overestimates the number of nerve fibers, leading to counting bias. The PGP9.5 protein used by Tey and Saffari seems to be a more specific marker for nerve fibers than S100 [24]. In addition to the above markers, other markers, such as βIII-tubulin and neurofilament, are also commonly used for the labeling of nerves [25,26], and the effectiveness of these markers for detecting nerves in the skin has seldom been reported.

In addition to changes in quantity, spatial and morphological alterations in nerve fibers can also be observed in keloids. First, nerve fibers in keloids are located deeper in the dermis than those in normal skin. In addition, the nerve fibers are more densely distributed at the fringe of keloids than at the center region [22], which may help explain why itching is more commonly experienced at the border region of keloids [27]. On the other hand, nerve fibers in keloids are thinner, probably due to the compression of the densely deposited extracellular matrix [20]. The lack of flexibility may result in increased traction of the peripheral nerves. This increased mechanical tension may cause chronic and consistent stimulation to regional nerve fibers, resulting in pain and itch.

Apart from the differences in nerve characteristics within individual keloids, prior studies have indicated that keloids originating from distinct anatomical locations demonstrate differences in tension, which may correlate with pain and pruritus [28,29]. These differences may be attributed to more pronounced inflammatory responses triggered by increased mechanical stretching in truncal areas or highly movable areas, such as the chest, neck, and epigastric regions [30]. These differences highlight how mechanical tension influences keloid pathology, inflammation, and symptom severity, suggesting that targeted therapies may be needed for keloids in high-tension areas to better manage symptoms [31].

However, Hochman’s conclusion that nerve fibers in keloids are thinner was based on light microscopy observations following immunohistochemical staining [20]. Currently, the assessment of nerve diameter predominantly relies on electron microscopy. Furthermore, recent advancements in nerve visualization techniques, such as whole-mount immunostaining and three-dimensional imaging [32,33,34], have been developed. However, these methods have not yet been applied to the study of nerve structures in keloids. Therefore, additional investigations are needed to explore the neuroanatomical changes in keloid tissue.

In addition to the anatomical alterations in nerve fibers, dysfunction of the sensory nerves has been reported in keloids. Lee et al. performed quantitative sensory testing (QST) on 28 keloid patients, most of whom had itching or pain [27]. There were significant differences in cold, warm, and heat pain sensation thresholds between keloid, perikeloid and normal skin, which indicates that there is small nerve fiber neuropathy in keloids [27].

QST is a common technique for diagnosing peripheral nerve injury and includes a wide range of tests, such as mechanical/vibration detection thresholds and mechanical/pressure pain thresholds [19,35]. However, in addition to thermal testing, no study has conducted comprehensive QST to evaluate the different types of nerve lesions in keloids [36]. In addition to QST, other sensory testing techniques, such as neuroimaging and wireless itch sensors, can be used to objectively evaluate keloid-related pruritus and further elucidate the underlying sensory activation patterns [37,38].

## 3. Neurogenic Inflammation

Neurogenic inflammation refers to the inflammation triggered by the release of substances, such as neuropeptides, following the stimulation and activation of the nervous system. This process is closely linked to the development of pain and pruritus [39]. In fact, during wound healing and scar formation, mechanical stimuli can activate peripheral nerve fibers, leading to the secretion of neuropeptides from the peripheral terminals of primary afferent sensory neurons. These neuropeptides, in turn, interact with immune cells, keratinocytes, fibroblasts, and other cell types [40]. As the wound gradually heals, neurogenic inflammation gradually subsides [41].

Ogawa et al. presented the hypothesis that neurogenic inflammation occurs during the formation of keloid scars [11]; they assumed that small-diameter primary afferent fibers are stimulated by mechanical stress, which leads to neurogenic inflammation. Researchers have shown that neuropeptides such as substance P (SP) and calcitonin gene-related peptide (CGRP) are expressed at higher levels in pathological scars than in normal skin, which may be related to the occurrence of pain and itch symptoms [42,43,44,45,46,47] (Table 1).

SP binds to the neurokinin-1 (NK1) receptor, which is widely expressed in the skin microenvironment. In rodent models, SP induces histamine release by mast cell degranulation. Additionally, SP binds to multiple types of immune cells, causing chemotactic effects on T cells, monocytes/macrophages, eosinophils, etc., and promoting the secretion of inflammation-related cytokines by these cells [48].

CGRP is often simultaneously released with SP from nociceptive fibers [49]. It induces the release of IL-1β and tumor necrosis factor (TNF)-α from macrophages, as well as the proliferation of lymphocytes and the inhibition of IL-2 expression [50]. Moreover, CGRP may regulate the presentation of antigens by LCs to T cells through actions on microvascular endothelial cells (ECs), resulting in an increased production of IL-6 and IL-17A and eventually inducing Th17-type inflammation [51].

However, rigorous correlation analysis in a large cohort of keloid patients is warranted to confirm the relationship between these neuropeptides and the degree of pain and itch. Additionally, there has been a growing focus on NK1 receptor antagonists as therapeutic targets for itchiness in inflammatory skin diseases [52]. However, to date, no studies have explored the use of neuropeptide inhibitors or their receptor antagonists in the treatment of keloids. This research gap presents an opportunity for future studies, as targeting neurogenic inflammation could offer a novel approach to alleviate the pain and pruritus commonly associated with keloid formation.

## 4. Dysfunction of Schwann Cells and Fibroblasts

### 4.1. Dysfunction of Schwann Cells During Keloid Formation

As the main glial cells in the peripheral nervous system, Schwann cells play a pivotal role in axonal ensheathment and nerve regeneration. However, the role of Schwann cells in keloid formation has been largely neglected.

In recent years, with the development of single-cell RNA sequencing (RNA-seq) techniques, studies have shown that there is a difference in the aggregation of Schwann cells in keloids compared to that in normal skin [5,53]. Generally, Schwann cells are classified into two main types: myelinating and nonmyelinating. However, a specific subset of Schwann cells has been found to be enriched in the superficial layer of the keloid dermis, where they align with collagen bundles rather than axons [5]. Further subtype analysis revealed that this particular group of Schwann cells (NES+, IGFBP3+, and IGFBP5+) exhibited a significantly increased proportion in keloids. Gene function enrichment analysis designated this group of Schwann cells as profibrotic cells. Moreover, the transcriptional gene expression landscape of profibrotic Schwann cells in keloids is similar to that of Schwann cells involved in the repair of nerve injury [53]. Normally, Schwann cells involved in repairs exist temporarily after nerve injury to functionally regenerate a disrupted nerve [54]. The persistent activation of Schwann cells in keloids may be responsible for the overactivation of extracellular matrix secretion, which exerts additional mechanical tension on regional nerve fibers, leading to pain and itch symptoms in keloid patients.

Recently, Abdo et al. reported that terminal Schwann cells located at the dermo-epidermal border (AQP1+ PLP+ SOX10+) are involved in the transmission of pain sensation [55,56]. This study opens a new pathway for explaining the high frequency of pain and itch in keloid patients; however, this field is still in its infancy and requires further exploration.

### 4.2. Overactivation of Fibroblasts and Excessive Collagen Deposition

Mechanical stimuli caused by compression are among the most common causes of peripheral nerve injury [57]. Fibroblast activation and collagen nodule formation in keloid scars may compress the nerve fibers, leading to nerve injury or neurogenic inflammation and eventually causing sensory abnormalities [20,58]. Fibroblast activation is intricately regulated by multiple cell types. For instance, a study on systemic sclerosis revealed that IL-25-primed keratinocytes can enhance fibrosis by favoring extracellular matrix deposition over degradation [59]. This suggests that other cell types may also contribute to neurological dysfunction by participating in the fibroblast activation and collagen deposition process.

Keloid scars arise from an abnormal wound healing process, which includes the regeneration of damaged nerve fibers. Studies have shown that the fibrotic response after nerve injury that causes nerve scarring is closely related to axonal regeneration and the function of regenerating nerves [60]. Excessive fibrosis can lead to endoneurial hypoxia, which affects nerve regeneration and leads to sensory abnormalities [61]. On this basis, inhibiting myofibroblast activation can promote nerve regeneration and improve the recovery of nerve function [62,63,64].

In recent years, researchers have identified a specific disease-causing subcluster of fibroblasts within keloids, known as mesenchymal fibroblasts, characterized by a high expression of POSTN and CTHRC1 [4,65]. POSTN has been reported to bind different kinds of integrins, inducing itch sensation via the direct stimulation of peripheral sensory nerve fibers and initiating the secretion of itch mediators via immune and nonimmune cells [66]. Notably, a distinct fibroblast subpopulation has been identified in keloids, which is clearly associated with inflammatory responses. These pro-inflammatory fibroblasts may be linked to the development of pain and pruritus in keloids, although further in-depth evidence is still needed to fully establish this connection [3,65,67,68]. CTHRC1 has been reported to be involved in the regulation of Schwann cell proliferation and migration through the Sox2ot/miR-9/CTHRC1 axis, which in turn plays a role in the peripheral nerve repair process [69,70]. Consequently, it is hypothesized that regenerated uninhibited nociceptive fibers transmit itch sensation within keloids, although direct evidence supporting this mechanism is currently lacking and requires further investigation. Thus, substantial research is still required before these molecules can be reliably used as diagnostic markers for identifying keloid patients at risk of nerve-related complications such as itch and pain. Similarly, only with more evidence supporting their roles in these processes can targeted therapies, such as inhibitors or antagonists to block their interaction with integrins or modulate Schwann cell behavior, be considered as viable therapeutic strategies.

## 5. Summary and Future Directions

Pain and pruritus in keloids are common and very important symptoms. However, the molecular mechanisms of pain and pruritus have not been elucidated, which has led to a paucity of clinical studies focusing on the relief of keloid pain and itching through medication.

The diagnosis of small-fiber neuropathy relies on abnormalities in QST and intraepidermal nerve fiber density (IENFD) [71]; these methods have been used in the diagnosis of a variety of dermatoses, such as atopic dermatitis and prurigo nodularis [72,73,74]. Given that studies have shown abnormalities in QST and IENFD in keloid patients, it is likely that small-fiber neuropathy is present within keloids, suggesting that reduced IENFD may occur within the lesions, especially painful/itchy lesions [18]. However, previous studies have reached conflicting conclusions regarding the abnormal number of nerve fibers in keloids, so further research is needed to determine whether there are differences in nerve fiber density in keloid skin compared to normal skin.

Although neurogenic inflammation has been revealed to be active in pathologic scarring, drugs that target the release of neuropeptides and their binding to receptors have not been investigated. A previous study suggested that botulinum toxin type A can relieve pain in patients with keloids [75], possibly through its inhibition of the release of inflammatory mediators such as SP and CGRP [76,77]; however, this topic has not been further investigated. In recent years, antagonists of NK1, the receptor for SP, have been extensively studied for their ability to relieve pain and pruritus [78,79]; CGRP antagonists have also been widely studied for migraine treatment, including a patch form for transdermal delivery [80]. However, efforts should be made to evaluate the effectiveness of these neuropeptide-targeting drugs for controlling pain and itch symptoms in keloid patients through clinical trials.

In conclusion, itching and pain are two hallmark symptoms in keloid patients, and the underlying mechanisms are complicated. Accumulating evidence suggests that these symptoms might be related to neurological dysfunction within keloids, including anatomical alterations and sensory disturbances in nerve fibers and the regional neurogenic inflammatory microenvironment (dysregulated neuropeptide release and dysfunction of Schwann cells and fibroblasts). The recognition that neurological dysfunction explains many of the symptoms in keloids according to the mechanistic data reviewed here opens new pathways for drug discovery.

## Figures and Tables

**Table 1:** Neuropeptides in pathological scars.

Author	Year	Disease	Neuropeptide	Techniques
Kwak I.S. et al. [42]	2014	HS	SP ↑ CGRP ↑	Immunohistochemical staining
Suarez E. et al. [43]	2015	HS	SP ↑ CGRP ↑	Scar tissue qRT-PCR
			CGRP ↑	Primary fibroblast qRT-PCR
			NPY ↑	Primary fibroblast Western blotting
		K	CGRP ↑	Scar tissue qRT-PCR
			SP ↑ CGRP ↑ NPY ↑	Primary fibroblast qRT-PCR
			SP ↑ CGRP ↑	Primary fibroblast Western blotting
Li C. et al. [44]	2021	HS	NPY ↑	GEO data analysis
		K		
Crowe R. et al. [45]	1994	HS	NPY ↑ VIP ↑ SP ↑ CGRP ↑	Immunofluorescence
Scott JR et al. [46]	2005	PS	SP ↑	Indirect enzyme-linked immunosorbent assay
		HS		
Aubert A et al. [47]	2025	HS	SP ↑	Immunostaining
		K		

HS: hypertrophic scar; K: keloid; PS: postburn scar; NPY: neuropeptide Y; VIP: vasoactive intestinal peptide; SP: substance P; CGRP: calcitonin gene-related peptide. ↑: upregulated.

## Data Availability

No data were used for the research described in the article.

## Abbreviations

The following abbreviations are used in this manuscript:

IL, interleukin; LCs, Langerhans cells; QST, quantitative sensory testing; SP, substance P; CGRP, calcitonin gene-related peptide; NK1, neurokinin-1; TNF-α, tumor necrosis factor α; Ecs, endothelial cells; IENFD, intraepidermal nerve fiber density; ECM, extracellular matrix; NPY, neuropeptide Y; VIP, vasoactive intestinal peptide; NGF, nerve growth factor; CCL, C-C motif chemokine ligand; CXCL, C-X-C motif chemokine ligand.
